# Surveillance of perchlorate in ground water, surface water and bottled water in Kerala, India

**DOI:** 10.1186/s40201-015-0213-z

**Published:** 2015-07-28

**Authors:** Anupama Vijaya Nadaraja, Prajeesh Gangadharan Puthiyaveettil, Krishnakumar Bhaskaran

**Affiliations:** Environmental Technology, CSIR-National Institute for Interdisciplinary Science & Technology, Thiruvananthapuram, 695019 India

**Keywords:** Ground water, Ion chromatography, Kerala, Perchlorate, Thyroid disorders

## Abstract

**Background:**

Perchlorate is an emerging water contaminant that disrupts normal functioning of human thyroid gland and poses serious threat to health, especially for pregnant women, fetus and children.

**Results:**

High level of perchlorate contamination in fresh water sources at places nearby ammonium perchlorate (rocket fuel) handled in bulk is reported in this study. Of 160 ground water samples analyzed from 27 locations in the State Kerala, 58 % had perchlorate above detection limit (2 μg/L) and the highest concentration observed was 7270 μg/L at Ernakulam district, this value is ~480 times higher than USEPA drinking water equivalent level (15 μg/L). Perchlorate was detected in all surface water samples analyzed (*n* = 10) and the highest value observed was 355 μg/L in Periyar river (a major river in the State). The bottled drinking water (*n* = 5) tested were free of perchlorate.

**Conclusions:**

The present study underlines the need for frequent screening of water sources for perchlorate contamination around places the chemical is handled in bulk. It will help to avoid human exposure to high levels of perchlorate.

## Background

Perchlorate (rocket fuel) is an oxyanion (ClO_4_^−^), extensively used in arms and ammunition industries [1]. The chemical is reported as a potential thyroid disruptor by inhibiting iodide uptake causing hypothyroidism and associated health effects especially in infants, pregnant women and foetuses [2, 3]. A number of animal studies have reported ClO_4_^−^ induced toxicities including delayed metamorphosis, haemolytic anaemia, thyroid tumor development etc. [4–6]. The current health advisory level for ClO_4_^−^ is set at 15 μg/L based on the reference dose recommended by US EPA [7]. The World Health Organization (WHO) established provisional maximum tolerable daily intake (PMTDI) of 0.01 mg/kg body weight for ClO_4_^−^ [8]. However, in many countries including India, drinking water/wastewater standard for ClO_4_^−^ is yet to be defined. Detailed assessment and continuous monitoring of ClO_4_^−^ in water sources have been reported from various countries such as USA [9], Canada [10], Europe and Middle East [11], Japan [12], Korea [13], India [14, 15] and China [16]. Perchlorate was detected in several food samples at concentrations above the Reference Dose (RfD) of 0.053 μg/kg bw/day proposed by National Academy of Sciences [17]. In USA, ClO_4_^−^ was detected in 39 infant formulas at concentrations ranging from <0.4–13.5 μg/L [18].

An earlier study has reported ClO_4_^−^ in 76 % of water samples collected from 13 locations in six States/Union territory (Tamilnadu, Karnataka, Bihar, Maharastra, West Bengal and Pondicherry) in India with concentrations ranged from <0.02–6.9 μg/L [14]. But, a more recent study has reported ClO_4_^−^ in the range <0.005–7,690 μg/L in ground water samples from near cracker manufacturing industrial area (Sivakashi) in Tamil Nadu, India [15]. Unlike the states covered in previous two studies, Kerala has two known major inventories of ClO_4_^−^. One is the ammonium perchlorate experimental plant (APEP) at Aluva in Ernakulam district where this chemical is produced in bulk and other place is Vikram Sarabhai Space Research Centre (VSSC) at Thumba, Trivandrum district. A preliminary study conducted in our lab (in 2010) has revealed wide contamination of ClO_4_^−^ in water samples from many districts in Kerala. In a different perspective, a study conducted in the coastal area of central Kerala revealed 10-15 % of iodine-sufficient population suffering from thyroid disorders [19]. Studies have also shown high incidence thyroid cancer in Kerala compared to major cities in India [20]. However, a proper reason for these serious health problems could not be identified yet. In view of this the present study focus on a detailed assessment of ClO_4_^−^ in ground and surface water samples giving emphasis to places where this chemical is handled in bulk. The finding of this study underlines the need for regular screening of ground and surface water sources for perchlorate around places where this toxic chemical is handled in bulk.

## Methods

### Study area

**Fig. 1:**
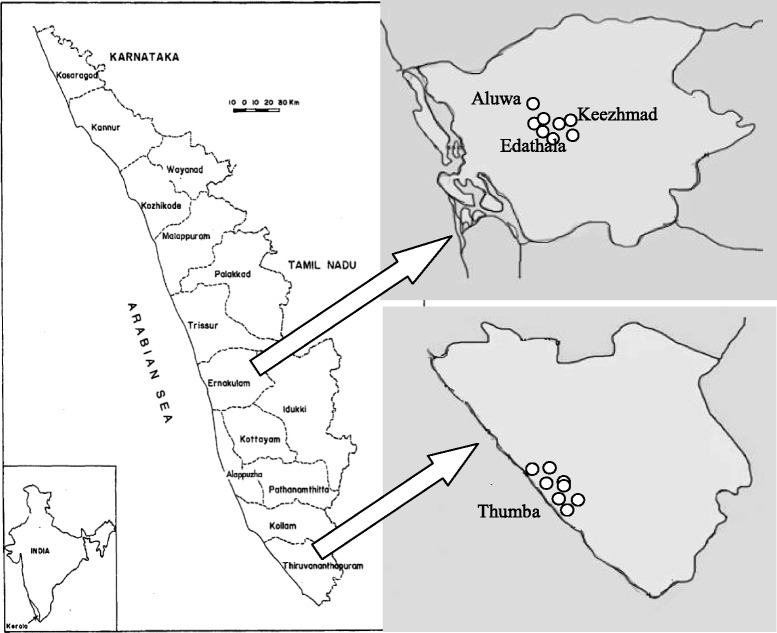
Area map showing sample collection sites in Thiruvananthapuram and Ernakulam districts in Kerala, India

Five sites in Kerala were selected in this study for detailed assessment of ClO_4_^−^ contamination. One of the sites was near Ammonium Perchlorate Experimental Plant (APEP) at Aluwa in Ernakulam district. The second site was at Thumba, near Vikram Sarabhai Space Centre (VSSC) in Thiruvananthapuram district. The other two locations were in Kannur and in Palakkad districts. Our preliminary study has revealed high ClO_4_^−^ level in water samples from these four districts. Location map of the study area is presented in Fig. 1. A fifth site, Koorg (latitude 12.33749° and longitude 75.80691°) in the nearby State Tamilnadu was selected as a control site. This is a hilly region (altitude ~ 5000 feet above sea level) and a far off place from any known inventories of perchlorate. Sampling was done from 27 locations in Kerala.

### Sampling

Sampling was done during March − May 2012. Hundred ml of sample was collected from each point and filtered using 0.2 μm filters (Millipore) and brought to laboratory and stored at 4 °C till analysis. Repeated sampling was done from sites that showed high ClO_4_^−^ values.

### Perchlorate analysis in commercial drinking water

Perchlorate was also analyzed in 5 brands of bottled drinking water available in local market. These brands include Kinley, Aquafina, Neyyar aqua, Green valley and Surabhi.

### Instrumental analysis

Water sample analysis was performed using an Ion Chromatography system (IC-1100, Dionex) with a separation column − Ion Pac AS 16 (2 × 250 mm and 4× 250 mm), guard column − Ion Pac AG 16 (2 × 50 mm and 4 × 50 mm) and an anion self-regenerating suppressor ASRS 300 (4 mm). The Ion Pac AS 16 column is specific for ClO_4_^−^ ion with a lower detection limit of 2 ppb (μg/L). This method for ClO_4_^−^ detection in drinking water is recommended according to USEPA methods 314.0 and 314.1. The eluent used was 50 mM Sodium hydroxide (NaOH, Fluka) at a flow rate 1.5 mL/min. The injection volume was 1000 μL. Calibration standards of ClO_4_^−^ was prepared with high purity KClO_4_ (Sigma Aldrich) by diluting 1000 mg/L primary standard. All solutions were prepared in ultra-pure milliQ water (Millipore).

### Quality assurance and quality control (QA/QC) for IC

Three sets of calibration curves were generated ranging from 5–30, 50–100 μg/L and 100–500 μg/L. Laboratory reagent blank and fortified samples were also analyzed for QC. The mean recovery of ClO_4_^−^ with the AS16 column and analytical condition was 100 ± 10 %.

## Results and discussion

### Detailed assessment of perchlorate in water samples

**Fig. 2:**
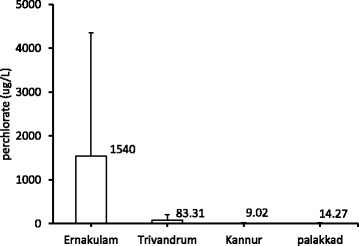
Perchlorate concentration (mean/SD) at different districts in Kerala

**Fig. 3:**
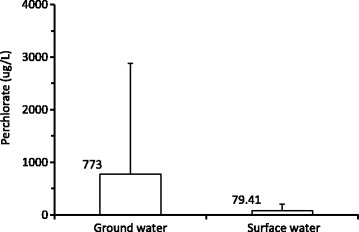
Perchlorate concentration (mean/SD) in groundwater and surface water samples in Kerala

The present detailed assessment of ClO_4_^−^ was based on our preliminary study in the past. We have observed ClO_4_^−^ contamination of water samples from public drinking, open well and surface water sources at few districts in Kerala and a maximum of 91.4 ppb ClO_4_^−^ was observed in open well water (for drinking) from Thumba (near VSSC) [21]. More detailed screening of samples was done during this study. Perchlorate concentration in ground and surface water samples collected from different locations in Kerala is presented in Table 1 and 2 respectively. Of the 160 samples analyzed from 27 locations, 58 % had ClO_4_^−^ above detection limit (2 μg/L). The highest concentration observed were 6420 μg/L and 7270 μg/L in samples from house hold open well at Edathala and from a public open well at Kulakkad respectively (both places are ~500 meters away from APEP). These values are ~480 times higher than drinking water equivalent level (DWEL), 15 μg/L established by USEPA. Perchlorate was detected in all the samples collected from Periyar river in the area with an average concentration 122 μg/L (*n* = 4) and the highest concentration observed was 355 μg/L. Periyar river (~3 km away from APEP) flows through the states of Tamil Nadu and Kerala, and it is one of the few perennial rivers in the region and provides drinking water to major town like Ernakulam in Kerala. Analysis of water samples from Trivandrum district also revealed high concentration of ClO_4_^−^. The highest value observed was 300 μg/L in a house hold open well water from Thumba (near VSSC). The average ClO_4_^−^ level in this region was 85 ± 45 μg/L (*n* = 7). The mean concentration of ClO_4_^−^ in ground water samples from all the study area is given in Fig. 2. Ground water perchlorate level was high in Ernakulam and low in Kannur district. Fig. 3 shows a comparison of perchlorate in all ground water and surface water sources studied. Compared to surface water (mean = 773), the ground water (mean = 79) perchlorate level is almost 10 times higher.

**Table 1:** Perchlorate (μg/L) in ground water samples from different districts in Kerala

District	Sampling site	No. of samples	Highest value	Mean value	Median value	Geometric mean
	Aluva town	4	BDL	BDL	BDL	BDL
	Keezhmad	5	2.76	0.52±1.2	BDL	1.22
	Marampalli	4	4.7	1.2±2.3	BDL	1.47
Ernakulam	Kulakkad site 1	7	666	243±218	185	170
	Kulakkad site 2	2	7270	7230±49.5	7230	7230
	Nalammile	15	667	243±278	45.4	28.3
	Edathala site 1	3	6420	4270±3700	6400	345.2
	Vazhakulam	5	6.74	1.67±2.9	BDL	1.6
	Edathala site 2	8	BDL	BDL	BDL	BDL
	Kazhakutom	10	7.07	4.05±1.2	3.8	3.9
	Manavila	7	10.9	6.46±5.2	4.83	3.9
Trivandrum	Kumapuram	12	9.19	3.66±4	2.8	2.66
	Thumba site 1	7	155	85±40	75	77.3
	Thumba site 2	2	300	300±80	294.7	294
	Veli	5	17.7	9.88±7.3	12.32	6.87
	Kannapuram	4	20.10	13.65±4.9	13.2	12.9
Kannur	Morazha	3	17	5.67±9.8	BDL	2.57
	Kannur town	4	BDL	BDL	BDL	BDL
	Thalassery	2	BDL	BDL	BDL	BDL
	Arayal	4	17	10.15±8.3	11.8	6.64
	Chittur	4	13.2	11.6±1.6	11.9	11.5
Palakkad	Palakkad town	5	15.0	14±1.02	13.6	13.9
	Olarakode	2	8.2	7.19±1.4	7.19	7.12
Koorg (control site)	Koorg	2	BDL	BDL	BLD	BLD

**Table 2:** Perchlorate (ug/L) in surface water samples from different districts in Kerala

District	Sampling site	No. of samples	Highest value	Average	Median	Geometric mean
Trivandrum	Veli lake	2	19.6	16.4	16.4	16
Ernakulam	Periyar river	4	355	122	35.4	66.8
Palakkad	Sokanashini river	2	21	17.5	15.75	17.14
Kannur	Vellikeel river	2	BDL	BDL	BDL	BDL

Perchlorate was detected in 60 % samples (*n* = 18) from various places in Kannur district with a highest value of 20.1 μg/L. Perchlorate was also detected in all the samples (*n* = 5) from Palakkad district with a peak value of 15 μg/L in sample collected from a rock mining site. As expected water samples from the public distribution system from Koorg had no detectable level of ClO_4_^−^ due its geographical location.

Reports on perchlorate contamination in drinking water supplies from different countries in recent years indicate the growing concern about ClO_4_^−^ contamination. Comparable to the high value (7270 μg/L) observed in this study, in a recent study 7,690 μg/ L ClO_4_^−^ was reported in ground water samples collected from fire cracker manufacturing sites Tamil Nadu, India [15].

USEPA has reported ClO_4_^−^ analysis data collected from 3,865 public water supplies between 2001 and 2005 from several states and territories. It was found that, ~4 % of samples had at least one analytical detection of ClO_4_^−^ at ≥4 μg/L and the maximum detected level was 420 μg/L.

### Probable sources of ClO_4_^−^ contamination in water sources in Kerala

The two inventories of ClO_4_^−^ could be considered as the major sources of its contamination in Ernakulam and Trivandrum districts. The NH_4_ClO_4_ manufacturing and using places in Ernakulam and Trivandrum districts respectively in this state could be one of the potential sources of ClO_4_^−^ contamination. Our preliminary study revealed high level of ClO_4_^−^ (91.4 μg/L) and chlorate (ClO_3_^−^) (177 μg/L) in water samples from Thumba, Trivandrum [21]. The present study also detected high level of ClO_4_^−^ (300 μg/L) from household well water from Thumba. However, unlike the previous study, the highest ClO_4_^−^ concentration (7270 μg/L) was observed in public well water source located in close proximity to APEP in Ernakulam district. Ammonium perchlorate washout from these sources can be the potential source of high level contamination detected in ground water in this region. Isotope Ratio Mass Spectra (IRMS) analysis will provide more concrete information about the source of perchlorate in this region and other places in Kerala. Moreover, assessment of thyroid gland functioning of people around APEP will bring out health effects due to the exposure to high level of ClO_4_^−^ in drinking water. Analyzing Thyroid gland functioning (TSH, T3, T4 etc. values) as well as Iodide and perchlorate level in urine samples will provide information about thyroid gland function and exposure level to perchlorate.

Perchlorate was also detected in water samples from the Northern districts, Kannur and Palakkad (20.11 and 15 μg/L) which are ~220 and ~150 km away from the APEP facility. The presence of ClO_4_^−^ in these regions points to other possible sources like usage of ClO_4_^−^ in fireworks, explosives etc. There are a large numbers of fire work display occurs in Kerala especially during April-May which is the festival season in the state. Several small scale fire work manufacturing units are operated during this period, however only few data about these industries are available as most of these are unauthorized. An increase in deposition of ClO_4_^−^ by 18 fold was observed due to fallout from fireworks in USA [22]. In another study, surface water ClO_4_^−^ level increase in the range of 24–1028 times was observed following fireworks display in Oklahoma Lake (USA) [23]. Perchlorate contamination due to firework production and display was also reported from China and Japan [12, 16]. As already mentioned, high levels of ClO_4_^−^ was detected recently from ground water samples from a cracker manufacturing industrial area in Tamil Nadu [15]. A number of rocks mine operating in most of the districts in Kerala. The explosives used in these mines may include ClO_4_^−^ salt also. Traces of ClO_4_^−^ from these sites may also contribute to ClO_4_^−^ contamination of waters in Kerala. This could be a potential source of ClO_4_^−^ in the water sample from near rocks mining areas of Palakkad. No information is available on the consumption of ClO_4_^−^ in rock mines, primarily due their unauthorized operational status similar to small scale fire-cracker industries. Perchlorate can also form naturally under rare environmental conditions like ozone oxidation of aqueous chloride or through electric discharging of chloride aerosol [24]. Natural ClO_4_^−^ deposits were found at relatively high concentration in Atacama Desert in Chile [25]. High concentration of ClO_4_^−^ (1000 mg/Kg) was detected in natural mineral ores like potash ore from places like New Mexico, Canada, Bolivia and California [26]. Stable isotope analysis of chlorine and oxygen in ClO_4_^−^ (^17^O/^16^O, ^18^O/^16^O and ^37^Cl/^35^Cl) can distinguish the nature of ClO_4_^−^ (synthetic or natural) present in environmental samples [27].

### Screening of commercial drinking water for perchlorate contamination

Perchlorate was not detected in any of the bottled drinking water samples tested in this study. Previous report from India also had a similar observation where ClO_4_^−^ was not detected in 5 of the branded of bottled drinking water analysed [14]. Perchlorate concentration was also very low (<0.002–0.22 μg/L) in bottled water from China [16]. However ClO_4_^−^ was found in 10 of the 21 bottled water samples in USA with a mean concentration of 0.16 μg/L [28].

## Conclusions

The present study reveals high level ClO_4_^−^ contamination in ground and surface water around places where ClO_4_^−^ is handled in bulk. The contamination was more severe in ground water (max. value 7270 μg/L) compared to surface water (max. value 355 μg/L), both from Aluva in Ernakulam district, Kerala. Findings of this study points to the need for frequent monitoring of ground water samples around places where ClO_4_^−^ is handled in bulk and necessitate epidemiological study in the contaminated area to assess the status of thyroid gland functioning. This study also underlines the need for defining water quality standards for perchlorate in India and also for controlling the environmental release of perchlorate especially from point sources like the manufacturing and using sites.

## References

[1] ITRC (Interstate Technology and Regulatory Council) (2005). Perchlorate: overview of issues, status, and remedial options.

[2] Wolff J (1998). Perchlorate and the thyroid gland. Pharmacol Rev.

[3] Henrichs J, Bongers-Schokking JJ, Schenk JJ, Ghassabian A (2010). Maternal thyroid function during early pregnancy and cognitive functioning in early childhood: the generation study. J Clin Endocrinol Metab.

[4] Stettler R (1977). Utilisation del’ozone dans le traitment de eaux de boisson. Gas Wass Abwass.

[5] Greer MA, Goodman G, Pleus RC, Greer SE (2002). Health effects assessment of environmental perchlorate contamination: The dose response for inhibition of thyroid radioiodine uptake in humans. Environ Health Perspect.

[6] Hu F, Sharma B, Mukhi S, Patino R, Carr JA (2006). The colloidal thyroxine (T_4_) ring as a novel biomarker of perchlorate exposure in the African clawed frog *Xenopus laevis*. Toxicol Sci.

[7] USEPA (United States Environmental Protection Agency) (2008). Interim drinking water health advisory for perchlorate.

[8] WHO (World Health Organization) (2010). Joint FAO/WHO expert committee on food additives seventy-second meeting.

[9] Hogue C (2003). Rocket-fueled river. Chem Eng News.

[10] Backus SM, Klawuun P, Brown S, D’sa I, Sharp S, Surette C, Williams DJ (2005). Determination of perchlorate in selected surface waters in the Great Lakes Basin by HPLC/MS/MS. Chemosphere.

[11] El Aribi H, Le Blanc YJC, Antonsen S, Sakuma T (2006). Analysis of perchlorate in foods and beverages by ion chromatography coupled with tandem mass spectrometry (IC-ESI-MS/MS*)*. Anal Chim Acta.

[12] Kosaka K, Asami M, Matsuoka Y, Kamoshita M, Kunikane S (2007). Occurrence of perchlorate in drinking water sources of metropolitan area in Japan. Water Res.

[13] Kim HK, Kim JH, Lee BC, Yu SJ, Kim HJ (2009). Occurrence of perchlorate in drinking water sources in Korea. Water Sci Technol.

[14] Kannan K, Praamsma ML, Oldi JF, Kunisue T, Sinha RK (2009). Occurrence of perchlorate in drinking water, ground water, surface water and human saliva from India. Chemosphere.

[15] Isobe T, Ogawa SP, Sugimoto R, Ramu K, Sudaryanto A, Malarvannan G (2013). Perchlorate contamination of groundwater from fireworks manufacturing area in South India. Environ Monit Assess.

[16] Wu Q, Zhang T, Sun H, Kannan K (2010). Perchlorate in tap water, ground water, surface waters and bottled water from China and its association with other inorganic anions and with disinfection byproducts. Arch Environ Contam Toxicol.

[17] NAS (National Academy of Science) (2005). Health implications of perchlorate ingestion.

[18] Wang Z, Lau BPY, Tague B, Sparling M, Forsyth D (2011). Determination of perchlorate in infant formula by isotope dilution ion chromatography/tandem mass spectrometry. Food Addit Contam.

[19] Usha MV, Sundaram KR, Unnikrishnan AG, Jayakumar RV, Nair V, Kumar H (2009). High prevalence of undetected thyroid disorders in an iodine sufficient adult south Indian population. J Indian Med Assoc.

[20] Jayalekshmi P, Gangadharan P, Mani KS (2006). Cancer in women in Kerala - a transition from a less-developed state. Asian Pac J Cancer Prev.

[21] Anupama VN, Kannan K, Prajeesh PVG, Rugmini S, Krishnakumar B (2012). Perchlorate, Chlorate and Bromate in water samples from the South-West coast of India. Water sci Technol-Water supply.

[22] Munster J, Hanson NG, Jackson AW, Rajagopalan S (2009). The fall out of fireworks: Perchlorate in total deposition. Water Air Soil Poll.

[23] Wilkin RT, Fine DD, Burnett NG (2007). Perchlorate behavior in municipal lake following fireworks display. Environ Sci Technol.

[24] Dasgupta PK, Martinelango PK, Jackson WA, Anderson TA, Tian K, Tock RW, Rajagopalan S (2005). The origin of naturally occurring perchlorate: Role of atmospheric processes. Environ Sci Technol.

[25] Ericksen GE (1983). The Chilean nitrate deposit. Sci Am.

[26] Orris GJ, Harvey GJ, Tsui DT, Eldrige JE (2003). Preliminary analyses for perchlorate in selected natural materials and their derivative products.

[27] Sturchio NC, Hoaglund JR, Marroquin RJ, Beloso AD, Heraty LJ, Bortz SE, Patterson TL (2012). Isotopic mapping of groundwater perchlorate plumes. Ground Water.

[28] Shi Y, Zhang P, Shi J, Cai Y, Mou S, Jiang G (2007). Perchlorate in sewage sludge, rice, bottled water and milk collected from different areas in China. Environ Int.

